# Supplementary material for: Is reporting quality in medical publications associated with biostatisticians as co-authors? A registered report protocol
Source: PLoS One. 2020 Nov 6;15(11):e0241897. doi: 10.1371/journal.pone.0241897 (PMC7647072; doi:10.1371/journal.pone.0241897)
Supplement: S1 Appendix — The file contains the search string for exposed and non-exposed publications for use in PubMed. (PDF) [file pone.0241897.s001.pdf]

S1 Appendix. Search string

Year 2017 ("2017.01.01"[Date - Publication] : "2017.12.31"[Date - Publication]) AND medline[sb] AND "english"[Language] AND hasabstract AND ("zurich"[Affiliation] OR "zuerich"[Affiliation] OR "zürich"[Affiliation]) NOT ("1900.01.01"[Date - Publication] : "2016.12.31"[Date - Publication])

Year 2018 ("2018.01.01"[Date - Publication] : "2018.12.31"[Date - Publication]) AND medline[sb] AND "english"[Language] AND hasabstract AND ("zurich"[Affiliation] OR "zuerich"[Affiliation] OR "zürich"[Affiliation]) NOT ("1900.01.01"[Date - Publication] : "2017.12.31"[Date - Publication])

Date when non-exposed publications on PubMed were assessed + list of all PMIDs: 2019-12-16

Date when exposed publications on PubMed were assessed: 2019-12-09
