# Supplementary material for: Is reporting quality in medical publications associated with biostatisticians as co-authors? A registered report protocol
Source: PLoS One. 2020 Nov 6;15(11):e0241897. doi: 10.1371/journal.pone.0241897 (PMC7647072; doi:10.1371/journal.pone.0241897)
Supplement: S1 File — The affiliation list was generated from the electronic search, with indication of included and excluded affiliations. (PDF) [file pone.0241897.s002.pdf]

Affiliation list

Affiliation	Frequency	Affiliation excluded
university of zurich	3672	
university hospital zurich	1409	
university children's hospital zurich	250	
university hospital of zurich	208	
university hospital zurich and university of zurich	98	
zurich university of applied sciences	97	x
university of zurich and eth zurich	86	
university hospital and university of zurich	60	
university and eth zurich	54	
university hospital of psychiatry zurich	38	
vetsuisse faculty university of zurich	32	x
zurich university hospital	30	
university and university hospital zurich	29	
eth zurich and university of zurich	24	
university heart center zurich	24	
zurich university of applied sciences (zhaw)	24	x
university children's hospital of zurich	22	
university of zurich-vetsuisse	20	x
university of zurich and university hospital zurich	19	
psychiatric hospital of the university of zurich	18	
university and university hospital of zurich	18	
university zurich	18	
university of zurich &	15	
university hospital zurich (usz)	12	
university of zurich and university hospital of zurich	11	
1 university of zurich	10	
zhaw zurich university of applied sciences	10	x
2 university of zurich	9	
eth and university of zurich	9	
university hospital for psychiatry zurich	9	
zurich university	9	
zurich university of applied sciences zhaw	8	x
university hospital zurich and city hospital waid	7	
university hospital zurich and clinical neuroscience center	7	
university hospital zurich (usz) and zurich university	6	
zurich university hospital and university of zurich	6	
university of zurich and department of health sciences and technology	5	
children's university hospital zurich	4	
department of chemistry university of zurich winterthurerstrasse 190	4	x
psychiatric university hospital zurich	4	
university hospital of zurich and university of zurich	4	
university hospital zurich &	4	
university of zurich (uzh)	4	
university of zurich and city hospital waid	4	
university of zurich and swiss federal institute of technology zurich	4	
university of zurich centre for travel medicine	4	
vetsuisse faculty of the university of zurich	4	x
1 university hospital zurich	3	
associated institute of the university of zurich	3	
o university of zurich	3	
universitatsspital zurich	3	
university children's hospital zurich and children's research center	3	
university clinic of zurich	3	

university heart centre zurich	3	
university of zuerich	3	
university of zurich and zurich center for integrative human physiology	3	
university of zurich hospital	3	
university of zurich uzh	3	
zurich university of applied science	3	x
2 university zurich	2	
a swiss research institute for public health and addiction at zurich university	2	
a universitatsspital zurich	2	
and university hospital zurich	2	
and university of zurich	2	
balgrist university hospital zurich	2	
clinical neuroscience center university hospital zurich zurich switzerland	2	
cteph programme university hospital zurich	2	
department of dermatology university hospital zurich	2	
division of infectious diseases and hospital epidemiology and children's research center university children's hospital zurich	2	
division of internal medicine university of zurich zurich switzerland	2	
eth university of zurich	2	
eth zurich and university of basel	2	x
ethz and university of zurich	2	
heart center university hospital of zurich	2	
institute of molecular cancer research of the university of zurich and eth zurich	2	
medical faculty of the university of zurich and department of health science and technology of the swiss federal institute of technology	2	
swiss federal institute of technology (ibz) eth zurich universitätstrasse	2	x
swiss research institute for public health and addiction associated with the university of zurich	2	
swiss research institute for public health and addiction at zurich university	2	
switzerland and university of zurich	2	
the university of zurich	2	
universitaetstrasse 2 - 8092 zurich	2	x
universitats-kinderspital zurich	2	
universitatsspital zurich klinik fur neuroradiologie	2	
universities of bern and zurich	2	
universities of berne and zurich	2	
university and eth zurich	2	
university children's hospital zurich and children's research centre	2	
university hospital and university of zurich (e.s	2	
university hospital and university zurich	2	
university hospital zuerich	2	
university hospital zurich usz	2	
university of applied sciences zurich	2	x
university of arts zurich	2	x
university of zurich (r.g	2	
university of zurich (uzh) and swiss federal institute of technology (eth)	2	
university of zurich and eth zurich . zurich	2	
university of zurich and eth zurich institute for biomedical engineering	2	
university of zurich and eth zurich zurich	2	
university of zurich and federal institute of technology (eth) zurich	2	
university of zurich and swiss federal institute of technology (eth zurich)	2	
university of zurich and swiss federal institute of technology in zurich	2	
university of zurich and university research priority program "	2	
university of zurich zurich	2	
university of zurich(switzerland)	2	
university zurich-irchel	2	

university zurich and university hospital zurich	2	
universityhospital zurich	2	
zuerich university of applied sciences (zhaw)	2	x
zurich university hospital of psychiatry	2	
zurich university of the arts	2	x
1 zurich university of applied sciences	1	x
2 institute of diagnostic and interventional radiology university hospital zurich	1	
a institute of occupational therapy at zurich university of applied sciences	1	x
a university of zurich	1	
§university of zurich	1	
and zurich university	1	
assistant professor of orthopaedics university of zurich	1	
associated with the university of zurich	1	
center for dental and oral medicine university of zurich	1	
center for dermatology and hair diseases professor trüeb and university of zurich	1	
clinic for geriatric medicine and centre on aging and mobility at the university of zurich	1	
clinical research priority program for rare diseases university of zurich	1	
clinical research priority program for rare diseases university of zurich"	1	
department of evolutionary biology and environmental studiesuniversity of zurich	1	
department of neurology university hospital zurich and university of zurich zurich switzerland	1	
department of neuroradiology university hospital zurich university of zurich zurich switzerland	1	
department of neurosurgery university hospital zurich university of zurich zurich switzerland	1	
division neuropsychology department of psychology university of zurich zurich switzerland	1	
division of stem cell transplantation university children's hospital zurich	1	
eidgenössische technische hochschule and university of zurich	1	
eth and university zurich	1	
from the psychiatric university hospital zurich (p.b.)	1	
institute of clinical chemistry university of zurich and university hospital of zurich	1	
institute university of zurich	1	
neuropsychology unit department of neurology university hospital zurich zurich switzerland	1	
physics department of the university of zurich	1	x
swiss federal institute of technology (eth) and university of zurich	1	
universitätsspital zurich	1	
universitätsstrasse 16 eth zurich	1	x
universitätsstrasse 8092 zurich	1	x
universitaetsspital zuerich	1	
university and balgrist hospital zurich	1	
university and eth zuerich	1	
university and eth zurich gloriastrasse 35	1	
university children's hospital zurich - eleonore foundation	1	
university children's hospital zurich and children's research center zurich	1	
university children's hospital zurich cdivision of clinical pathology	1	
university heart center zurich. susanne.markendorf@usz.ch	1	
university hospital balgrist and university hospital of zurich	1	
university hospital of zurich and center for molecular cardiology	1	
university hospital of zurich einstitute of medical virology	1	
university hospital zurich #medical practice hottingen	1	
university hospital zurich (bbs	1	
university hospital zurich [usz] and university of zurich	1	
university hospital zurich † division of gastroenterology and hepatology	1	

university hospital zurich and university of zurich ddepartment of medicine	1	
university hospital zurich and university zurich	1	
university hospital zurich bcenter of competence multimorbidity cuniversity research priority program "	1	
university hospital zurich binstitute of medical virology	1	
university hospital zurich buniversity of zurich chorten centre for patient oriented research and knowledge transfer	1	
university hospital zurich ccenter for microscopy and image analysis	1	
university hospital zurich dinstitute of medical microbiology eswiss national center for mycobacteria	1	
university hospital zurich duniversity of zurich	1	
university hospital zurich edivision of gerontopsychology and gerontology	1	
university hsopital zurich	1	
university of applied sciences of special needs education zurich	1	x
university of zurich-irchel	1	
university of zurich - vetsuisse	1	x
university of zurich and eidgenössische technische hochschule (eth) zurich	1	
university of zurich and eth	1	
university of zurich and federal institute of technology	1	
university of zurich and the eth zurich	1	
university of zurich bdepartment of nephrology	1	
university of zurich bnational center of competence in research	1	
university of zurich ccancer registry zurich and zug	1	
university of zurich cdepartment of gastroenterology	1	
university of zurich ddepartment of dermatology	1	
university of zurich dental school	1	
university of zurich departments of §psychiatry **neurology	1	
university of zurich fdepartment of pulmonology	1	
university of zurichjinstitute of medical virology	1	
zhaw (zurich university of applied sciences) school of social work	1	x
zurich center for integrative human physiology (zihp) university of zurichzurich	1	
zurich university ‡zurich center for integrative human physiology (zihp)	1	
zurich university children's hospital	1	
zurich university for applied sciences	1	x
zurich university for applied sciences (zhaw)	1	x
zurich university hospital for psychiatry	1	
zurich university of applied sciences (zuas)	1	x
zurich university of teacher education	1	x
