# Supplementary material for: Is reporting quality in medical publications associated with biostatisticians as co-authors? A registered report protocol
Source: PLoS One. 2020 Nov 6;15(11):e0241897. doi: 10.1371/journal.pone.0241897 (PMC7647072; doi:10.1371/journal.pone.0241897)

Variables

CONSORT

(6a) *Completely defined pre-specified primary and secondary outcome measures, including how and when they were assessed.*

In the methods and results section:

(i) Are all outcome measures, which are reported on in the results section, explicitly defined as main/primary or secondary outcome (if applicable) in the methods section?

Note that the words primary and secondary need to be used. Also the word outcome or an appropriate alternative needs to be used, possible terms are endpoint, response, variable of interest or target variable.

(ii) For each outcome measure, is it declared how they were assessed?

(iii) For each outcome measure, is it declared when they were assessed?

Category A: Yes to (i) AND yes to (ii) AND yes to (iii)

Category B: Yes to (i) AND ((yes to (ii) AND no to (iii)) OR (no to (ii) AND yes to (iii))

Category C: No to (i) OR (yes to (i) AND no to (ii) AND no to (iii))

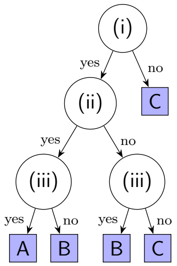

STROBE

(7) *Clearly define all outcomes, exposures, predictors, potential confounders, and effect modifiers. Give diagnostic criteria, if applicable.*

In the methods and results section:

(i) Are all outcomes, which are reported on in the results section, named and defined in the methods section?

Note that the word outcome or an appropriate alternative needs to be used, possible terms are endpoint, variable of interest or target variable.

(ii) Are all other variables, which are reported on in the results section, named and defined in the methods section? This includes exposures, predictors, potential confounders, potential effect modifiers etc.

Category A: Yes to (i) AND yes to (ii)

Category B: Yes to (i) AND no to (ii)

Category C: No to (i)

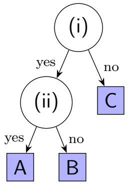

TRIPOD

(6a) *Clearly define the outcome that is predicted by the prediction model, including how and when assessed. [D;V]*

(7a) *Clearly define all predictors used in developing the multivariable prediction model, including how and when they were measured. [D;V]*

In the methods and results section:

(i) Are all outcomes, which are predicted by the prediction model, defined in the methods section?

Note that the word outcome or an appropriate alternative needs to be used, possible terms are endpoint, variable of interest or target variable.

Example (Diagnosis): Definite infection was defined by measurements taking values $\geq x$. Probable infection was defined by measurements taking values $\geq y$ $(x > y)$.

Example (Prognosis): Outcomes of interest were any type of death, disease related death, and disease related events.

(ii) Are all predictors, which are used in the prediction model, listed in the methods section?

(iii) Is it reported when the outcomes were measured?

In case of a prognostic model, this includes the specification of the timepoints, during the follow-up period, at which the outcomes were measured.

In case of a survival model, this requires the date of last follow-up.

(iv) Are for all predictors the units of measurements (continuous) or all categories (categorical/ordinal) specified?

(v) Is it specified when each predictor was measured?

Category A: Yes to (i) AND yes to (ii) AND yes to (iii) AND yes to (iv) AND yes to (v)

Category B: Yes to (i) AND yes to (ii) AND (yes to 1 or 2 out of (iii), (iv), (v))

Category C: No to (i) OR (yes to (i) and no to (ii)) OR (yes to (i) AND yes to (ii) AND no to (iii) AND no to (iv) AND no to (v))

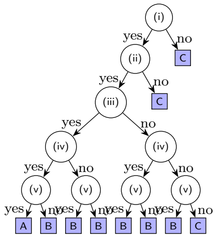

Study size

CONSORT

(7a) *How sample size was determined.*

In the methods section:

(i) Is a clear declaration of the target (i.e. a number known before data collection) sample size given?

(ii) It is mentioned on which primary outcome the calculation was based?

(iii) Is the method of sample size calculation given and does it fit the type of endpoint and does it fit the study design?

If the study design is not a common parallel group or a cross-over design, please answer all questions to the best of your knowledge and alert the coordination group.

(iv) Are the significance level (α, type I error) and the the statistical power (β, type II error) reported?

Category A: Yes to all questions

Category B: Yes to 2 - 3 out of the 4 questions

Category C: Yes to 1 or none out of the 4 questions

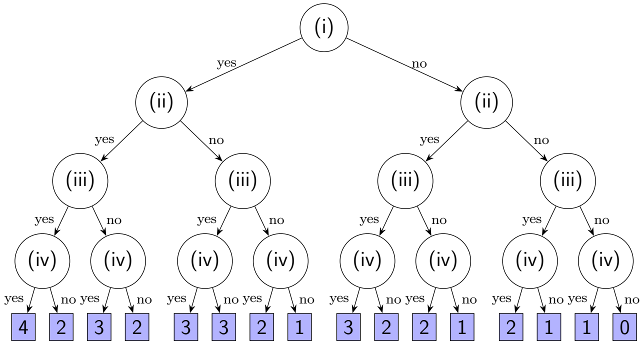

STROBE

(10) *Explain how the study size was arrived at.*

In the methods section:

(i) Have the authors refrained from post hoc justification for the study size and from retrospective power calculations?

(ii) Is a clear declaration of the target sample size given (i.e. a number known before data collection)?

(iii) Has a method of sample size calculation been used or is a description of the considerations that determined the sample size given?

Example for a description: The number of cases is determined by being all consecutive patients at a specific center in a specific time frame satisfying a specific set of criteria.

(iv) Is it mentioned on which outcome the sample size determination was based on?

Category A: Yes to (i) AND yes to (ii) AND yes to (iii) AND yes to (iv)

Category B: Yes to (i) AND Yes to (ii) AND ((no to (ii) AND yes to (iii)) OR (yes to (ii) AND no to (iii)))

Category C: No to (i) OR no to (ii) OR (yes to (i) AND yes to (ii) AND no to (iii) AND no to (iv))

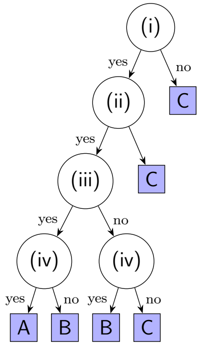

TRIPOD

(8) *Explain how the study size was arrived at. [D;V]*

In the methods section:

(i) Is a clear declaration of the target sample size given (i.e. a number known before data collection)?

(ii) Has a method of sample size calculation been used or is a description of the considerations that determined the sample size given?

Example for a description: The number of cases is determined by being all consecutive patients at a specific center in a specific time frame satisfying a specific set of criteria.

(iii) Is it mentioned on which outcome the sample size determination was based on?

In the methods or the discussion section:

(iv) Is the adequacy of the sample size in relation to the number of predictors used in the prediction model discussed?

Category A: Yes to (i) AND yes to (ii) AND yes to(iii) AND yes to (iv)

Category B: (Yes to (i) AND yes to (ii) AND ((yes to (iii) AND no to (iv)) OR (no to (iii) AND yes to (iv)))) OR (yes to (i) AND no to (ii) AND yes to (iii) AND yes to (iv))

Category C: (Yes to (i) AND yes to (ii) AND no to (iii) AND no to (iv)) OR (yes to (i) AND no to (ii) AND yes to (iii) AND no to (iv)) OR (yes to (i) AND no to (ii) AND no to (iii)) OR No to (i)

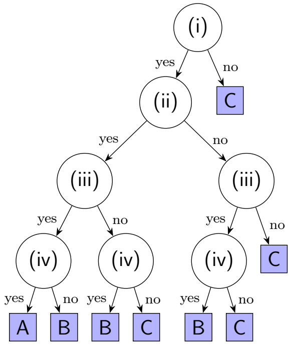

Missing data

CONSORT

(16) *For each group, number of participants (denominator) included in each analysis and whether the analysis was by original assigned groups.*

In the results section:

(i) For each analysis of the primary outcome(s) is it clearly specified on which population, e.g. intention-to-treat or per protocol etc., it is based?

(ii) Is the number of participants per intervention group reported for each analysis?

(iii) If no intention-to-treat population has been used in the analysis are the disadvantages of such an approach clearly stated? Answer yes if an intention-to-treat population has been used exclusively or in combination with other types of populations. Answer no if it is not clear which population has been used.

Category A: Yes to (i) AND yes to (ii) AND yes to (iii)

Category B: Yes to (i) AND ((yes to (ii) AND no to (iii)) OR (no to (ii) AND yes to (iii)))

Category C: (Yes to (i) AND no to (ii) AND no to (iii)) OR no to (i)

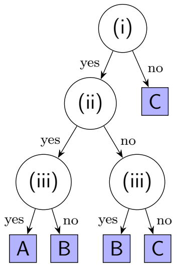

STROBE

(12c) *Explain how missing data were addressed.*

(14b) *Indicate the number of participants with missing data for each variable of interest.*

In the methods and results section:

(i) Are the numbers of missing values stated for each variable used in the primary analysis including the outcome? Answer yes to this question if no missing values have occurred.

(ii) Are the reasons for (non)-missingness stated? Answer yes to this question if no missing values have occurred and it is explained why they did not occur.

Example: the design allows no missing values or records with missing values are mentioned to be excluded

(iii) Have methods to impute missing values been specified and used? Answer yes to this question if no missing values have occurred.

(iv) Have results obtained using imputed data been compared to results obtained using a complete case analysis? Answer yes to this question if no missing values have occurred.

Category A: Yes to (i) AND yes to (ii) AND yes to (iii) AND yes to (iv)

Category B: (Yes to (i) AND yes to (ii) AND yes to (iii) AND no to (iv)) OR (no to (i) AND yes to (ii) AND yes to (iii) AND yes to (iv))

Category C: No to (ii) OR no to (iii) OR (no to (i) AND yes to (ii) AND yes to (iii) and no to (iv))

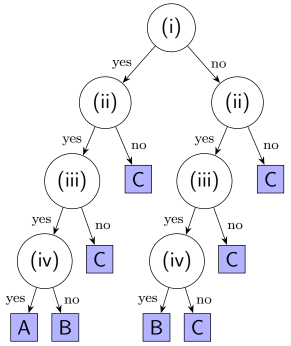

TRIPOD

(12c) *Explain how missing data were addressed.*

(14b) *Indicate the number of participants with missing data for each variable of interest.*

In the methods and results section:

(i) Are the numbers of missing values stated for each variable used in the primary analysis including the outcome? Answer yes to this question if no missing values have occurred.

(ii) Are the reasons for (non)-missingness stated? Answer yes to this question if no missing values have occurred and it is explained why they did not occur.

Example: the design allows no missing values or records with missing values are mentioned to be excluded.

(iii) Have methods to impute missing values been specified and used? Answer yes to this question if no missing values have occurred.

(iv) Have results obtained using imputed data been compared to results obtained using a complete case analysis? Answer yes to this question if no missing values have occurred.

Category A: Yes to (i) AND yes to (ii) AND yes to (iii) AND yes to (iv)

Category B: (Yes to (i) AND yes to (ii) AND yes to (iii) AND no to (iv)) OR (no to (i) AND yes to (ii) AND yes to (iii) AND yes to (iv))

Category C: No to (ii) OR no to (iii) OR (no to (i) AND yes to (ii) AND yes to (iii) and no to (iv))

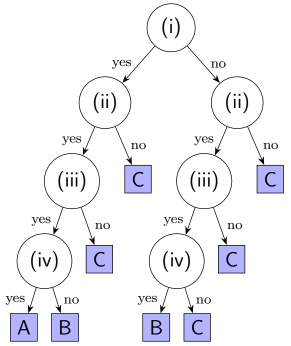

Statistical methods

CONSORT

(12a) *Statistical methods used to compare groups for primary and secondary outcomes.*

In the methods and results section:

(i) Is the statistical method used for the analysis of the primary outcome specified in enough detail such that it could be reproduced? This includes, for example, confidence level, two- or one-sided tests, treatment of unequal variances etc.

(ii) Are the results concerning the primary outcome that are reported in the results section clearly obtained by the method described in the methods section for the analysis of the primary outcome? If you answered no to (i), answer no to this questions as well.

(iii) If any secondary outcomes are specified, are the methods for their analysis stated in the methods section for each outcome or for each type of outcome? Answer yes to this question if no secondary outcomes are specified.

Category A: Yes to (i) AND yes to (ii) AND yes to (iii)

Category B: Yes to (i) AND ((yes to (ii) AND no to (iii)) OR (no to (ii) AND yes to (iii)))

Category C: (Yes to (i) AND no to (ii) AND no to (iii)) OR no to (i)

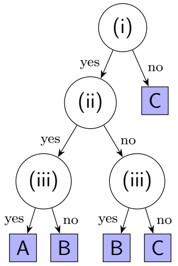

STROBE

(9) *Describe any efforts to address potential sources of bias.*

(12a) *Describe all statistical methods, including those used to control for confounding.*

In the results or discussion section:

(i) Was the potential for bias discussed in any way?

In the methods section:

(ii) Is it clearly stated which variables have been used in the adjustment?

(iii) Is it clearly stated how it has been determined which variables are used in the adjustment?

(iv) Is a protocol or an analysis plan or something similar mentioned and are exploraty/unplanned analyses clearly distinguished?

Category A: Yes to all questions

Category B: Yes to 3 out of the 4 questions

Category C: Yes to less than 3 questions of the 4 questions

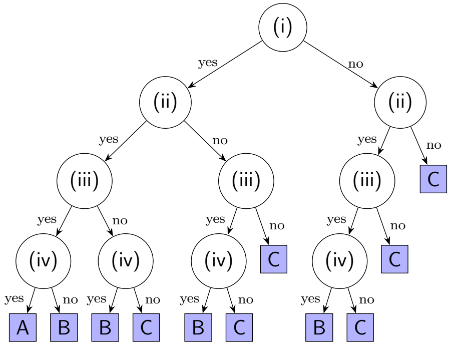

TRIPOD

(10d) *Specify all measures used to assess model performance and, if relevant, to compare multiple models*

In the methods section:

(i) Have measures to quantify calibration for the prediction model been specified?

Note that the word calibration does not have to be used.

(ii) Have measures to quantify discrimination of the prediction model been specified?

Note that the word discrimination does not have to be used.

In the discussion section:

(iii) Has the performance of the prediction model been compared to existing models? If the authors argue coherently that no other models exist, answer yes to this question.

Category A: Yes to (i) AND yes to (ii) AND yes to (iii)

Category B: Yes to (i) AND yes to (ii) AND no to (iii)

Category C: No to (i) OR no to (ii)

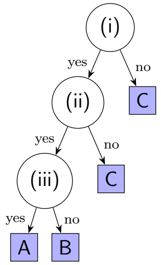

Results with precision

CONSORT

(17a) *For each primary and secondary outcome, results for each group, and the estimated effect size and its precision (such as 95% confidence interval).*

(17b) *For binary outcomes, presentation of both absolute and relative effect sizes is recommended.*

In the results section:

(i) Is the estimated between group difference for each primary outcome reported? If the primary outcome or one of the primary outcomes is binary: these are absolute estimated effect sizes.

(ii) If the primary outcome or one of the primary outcomes is binary: are relative estimated effect sizes reported? Answer yes to this question if none of the primary outcomes is binary.

(iii) Is the confidence interval for the estimated between group difference of each primary outcome reported?

(iv) If the primary outcome or one of the primary outcomes is binary: are for the relative estimated effect sizes also confidence intervals reported? Answer yes to this question if none of the primary outcomes is binary.

Category A: Yes to (i) AND yes to (ii) AND yes to (iii) AND yes to (iv)

Category B: Yes to (i) AND Yes to (ii) AND ((no to (ii) AND yes to (iii)) OR (yes to (ii) AND no to (iii)))

Category C: No to (i) OR no to (ii) OR (yes to (i) AND yes to (ii) AND no to (iii) AND no to (iv))

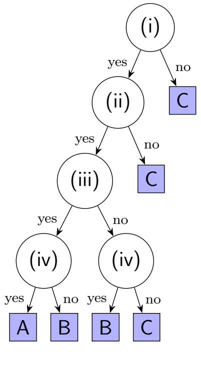

STROBE

(16a) *Give unadjusted estimates and, if applicable, confounder-adjusted estimates and their precision (e.g., 95% confidence intervals). Make clear which confounders were adjusted for and why they were included.*

In the methods section:

(i) Has it been declared and justified that no adjustment for confounding is necessary?

In the results section:

(ii) Are the unadjusted results for all of the point estimates reported?

(iii) Are the confidence intervals for all of the unadjusted point estimates reported?

(iv) Are the adjusted results for all of the point estimates reported?

(v) Are the confidence intervals for all of the adjusted point estimates reported?

Category A: (Yes to (i) AND yes to (ii) AND yes to (iii)) OR (no to (i) AND yes to (ii) AND yes to (iii) AND yes to (iv) AND yes to (v))

Category B: (Yes to (i) AND yes to (ii) AND no to (iii)) OR (no to (i) AND yes to two or three of (ii), (iii), (iv), (v))

Category C: (Yes to (i) AND no to (ii) AND no to (iii)) OR (no to (i) AND yes to one or none of (ii), (iii), (iv), (v))

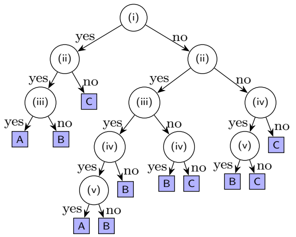

TRIPOD

(16) *Report performance measures (with CIs) for the prediction model. [D;V]*

In the results section:

(i) Are all performance measures which are described in the methods section reported in the results section for each of the proposed prediction models? If no performance measure is described throughout the paper answer no to this question.

(ii) Are all performance measures which are reported in the results section described in the methods section?

(iii) Are the confidence intervals or the p-values (e.g. 0.061 or < .001) for each reported performance measure reported in the results section?

Category A: Yes to (i) AND yes to (ii) AND yes to (iii)

Category B: Yes to (i) AND yes to (ii) AND no to (iii)

Category C: No to (i) OR no to (ii)

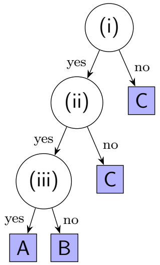

Supplement: S2 File — The questionnaire is intended to be used for rating the reporting quality in each study type. (PDF) [file pone.0241897.s003.pdf]
