# Supplementary material for: Is reporting quality in medical publications associated with biostatisticians as co-authors? A registered report protocol
Source: PLoS One. 2020 Nov 6;15(11):e0241897. doi: 10.1371/journal.pone.0241897 (PMC7647072; doi:10.1371/journal.pone.0241897)
Supplement: Attachment — Submitted filename: response.pdf [file pone.0241897.s004.pdf]

University of Zurich[UZH]

Department of Biostatistics

University of Zurich
Epidemiology, Biostatistics and Prevention Institute
Department of Biostatistics
Hirschengraben 84
CH-8001 Zurich

UZH, EBPI, Hirschengraben 84, CH-8001 Zurich

Dr. Ulrike Held
Head Working Group Clinical Biostatistics

Phone +41 44 634 45 39
Ulrike.Held@uzh.ch

Zurich, 10-05-2020

Cover Letter and Response to Reviewer Comments

Dear Managing Editor

Thank you for the careful review of our registered report protocol. We are very grateful for the constructive reviewer comments and believe that we could improve the manuscript by incorporating them. Our replies can be found from page 2 of this document.
We would also like to address the following questions:

Why is this publication useful for publication in PLoS ONE?
PLoS ONE announced the possibility of preregistration article types, the Registered Reports, in January 2020 (https://everyone.plos.org/2020/01/14/registered-reports-are-coming-to-plos-one/). The peer review of this study at the design phase emphasizes the importance of research protocols and the need for rigorous scientific practice in general. While we are concentrating on the biomedical literature in this article, we believe that the results may be interesting beyond biomedicine and hope to reach a wider audience with the publication at PLoS ONE.

How does this paper provide a worthwhile addition to the scientific literature?
There has been ongoing discussion about the methodological quality of medical research. Biostatisticians as members of research teams will add quality to study design and analysis, as well as reporting. To our knowledge, there has been no previous attempt to measure the impact of biostatisticians as collaborators in medical research.

How does this paper relate to previously published work?
There have been ongoing efforts to increase methodological knowledge among clinicians, such as the Statistics Notes by Doug Altman and co-authors in the British Medical Journal. Our study will raise awareness for use of reporting guidelines, and the value of biostatisticians as collaborators.

Which types of scientists do you believe will be most interested in this study?
The research is relevant for clinicians with active participation in medical research. Besides that, the field of meta-research has grown in recent years, aiming to develop strategies for research improvement. For biostatisticians, the research is relevant as is emphasizes not only the importance of sound design and analysis, but also the transfer of these aspects into the manuscript, while adhering to reporting guidelines.

Yours sincerely, on behalf of all authors,

Ulrike Held

Universität Zürich[UZH]

Response to reviewer comments for manuscript PONE-D-20-21077

Is reporting quality in medical publications associated with biostatisticians as co-authors? A registered report protocol.

We would like to thank all reviewers for their thorough review, encouraging comments and suggestions.

Reviewer 1

1) Your use of 'case' and 'control' is non-standard and may confuse some readers. This is the distinction that is used in observational studies where sampling is by outcome. Sometimes these are called retrospective studies but more commonly the preferable term 'case-control' study is used. For example, you might sample cases of lung-cancer and controls who do not have lung cancer. Other types of study classify groups by exposure, not by outcome. For example, you might classify subjects as exposed 'smoker' or not-exposed 'non-smoker'. Such studies are referred to as cohort studies. It is a standard epidemiological teaching that case control studies are good at studying multiple putative causal factors and cohort studies are good at studying multiple possible effects. Here you have the analogue of a cohort study, albeit with papers rather than subjects as the unit of study. I think it would be more usual to refer to the two groups as either exposed or non-exposed (as in an observational study) or 'treated' or possibly 'experimental' versus 'control' as for an interventional study. I agree that this terminology is not ideal here but I worry about what you have chosen. Perhaps all you need to note at the beginning is that 'case' and 'control' are not being used as in many observational studies.

Reply:
Thank you for pointing out the potentially confusing use of case and control in the context of our study. We agree, that in our study, the better wording would be “exposed” and “non-exposed”. We changed the wording throughout the registered report protocol, including figures, from case to exposed and from control to non-exposed.

2) I have checked your sample size calculation and find it to be correct provided that you intend to use a two-sample t-test with equal variances to perform a conventional significance test. Is this what you intend? You don't actually say what the intended analysis corresponding to your sample size calculation is.

Reply:
Thank you for pointing this out. We changed the paragraph for sample size calculation and it reads now as follows:
“With the given sample size of 95 exposed and 95 non-exposed publications in 2017 and 2018, at a significance level of 5% and with a power of 80%, an effect size of 0.41 (Cohen’s d) could be detected using a two-sample two-sided t-test with equal variances. The effect size would be considered a medium effect size.”

3) Later you refer to t-tests and Wilcoxon test and say they will be used 'as appropriate' but you don't actually say how you will decide what is appropriate. (Note I am definitely not encouraging you to use a test of

Normality to decide between them. Nevertheless this sort of 'as appropriate' statement is something that would not generally be accepted as adequate for a statistical analysis plan in drug development.)

Reply:
Thank you, we clarified that we intend to use the t-test or the Wilcoxon test, as appropriate based on visual inspection of histograms and QQ-plots. This was added to the statistical methods section.

4) Various typos and minor issues
a) L40 'Doug' not 'Dough'
b)L41 This quotation does not sound quite right. Please check it and provide a reference.
c)L50. Suggest 'most frequently used' rather than 'most frequent'
d) L120. 'TRIPOD' not 'TROPID'
e) L158 0.41 is a rather unusual choice of effect size. Is it the case that this is the value that guarantees 80% power for the sample size you intend to use?
f) L179. How will the confidence intervals be calculated? T-test. Hodges-Lehmann Wilcoxon

Reply:
We thank the reviewer for thorough review, all corrections were made. Regarding the effect size: we calculated the minimum detectable effect size, given the numbers of exposed and non-exposed papers in the corresponding years of publication. The confidence intervals will be calculated based on the t-distribution and assuming equal variances or with Hodges-Lehman Wilcoxon, depending on the distribution of the outcome. For that, data will be visualized with histograms and QQ-plots.
The following text was added to the statistical methods section:
"Confidence intervals will be calculated based on the t-distribution assuming equal variances or with Hodges-Lehman estimate based on the Wilcoxon test, corresponding to the statistical test chosen for each outcome."

Reviewer 3
This is a novel and important research idea that is much needed. As statistical techniques increase in sophistication, somewhat due to increased computing power, we can't expect physicians and practitioners to always know every statistical nuance that could be applied to their research. Reliance on statisticians to guide methodological decision-making should be normalized to advance science in the best, most sophisticated way.

Reply:
We thank the reviewer for this comment.

1. Consider re-labeling the "control" publications group as a "comparison" publications group. Since biostatisticians were not randomly assigned to author teams / projects, it is not a true control, but rather a comparison.

Reply:
Thank you for pointing this out, and we obtained a related comment from Reviewer 1. After careful consideration of cohort-study terminology, which is the study type that is most closely related to our research, we decided to use the words "exposed" and "non-exposed" publications, and we changed the terminology throughout the protocol and figures. We hope that Reviewer 3 would agree to this.

2. In the introductory paragraph that describes the comparison / control group, please be explicitly clear that this group is of papers who do not have a statistician listed as an author. This is implied in the introduction, but it could be clearer in this paragraph. (Lines 84-90, page 5).

Reply:
Thank you for this comment. We changed the wording of the paragraph and it reads now as follows:
"To define the group of "non-exposed" publications for comparison, all medical research publications found in PubMed between 2017 and 2018, with affiliation University of Zurich (UZH) or University Hospital Zurich (USZ) or any of the affiliated university hospitals for first and / or second author were extracted on Dec 16, 2019 (Supporting File 1). The non-exposed publications do not have any of the biostatisticians of our group on the author list."

3. One item to discuss or acknowledge is whether the research center from which you pull these papers has an authorship policy of some sort that would impact whether a biostatistician is added to the authorship list. For example, is there a standard policy of deciding authorship on papers? If yes or no, please describe. Also, is there an informal or collegial policy that this research center follows with regards to authorship? For example, if you know that statisticians are always included as authors when they help with methodologies in this research center, that would be good to state in this report protocol as a certainty that the study cases will be correctly classified.

Reply:
Thank you for this important comment. We reviewed the authorship guidelines from University of Zurich, referring to the guidelines by Swiss Academies of Arts and Sciences. There, the authorship entitlement is based on the following criteria, which we added to the introduction section.
"Authorship guidelines at our center (University of Zurich, Switzerland) are based on fulfilling the criteria of Swiss Academies of Arts and Sciences, by (1) making a substantial contribution to the planning, execution, evaluation and supervision of research; (2) involvement in writing the manuscript; and (3) approving the final version of the manuscript."

4. Another thought, that may or may not be relevant, but you may wish to address – are statisticians sometimes acknowledged or thanked in papers but not added as authors? If they are thanked and were a part of the research, but not an author on the manuscript which is then classified in the control group, this may represent a mis-classification of that paper.

Reply:
Thank you for this important comment. Indeed, we as statisticians are sometimes asked to be mentioned in the acknowledgements section. In our group, we dislike this because only by being a co-author we can take the responsibility to write parts of the manuscript, critically revise the manuscript, and approve the final version. Our high standards regarding reproducibility and properness of statistical analysis and reporting of results can only be guaranteed to transfer into the manuscript with us being co-authors. Therefore, we do not really fear mis-classification of papers into the comparison group.
We added to the introduction section:
"Sometimes, a collaborating biostatistician is mentioned in the acknowledgements section. However, only by being a co-author the biostatistician can take the responsibility to write parts of the manuscript (e.g. statistical methods, results), critically revise the manuscript, and approve the final version."

And further down to the discussion section:
"Finally, there is potential for misclassification of publications with a biostatistician responsible for the analysis, but with a mention only in the acknowledgements. However, in such situations, the biostatistician would not get the chance to critically revise the manuscript and to approve the final version – which could nevertheless lead to low reporting quality or even false interpretations of results."

Reviewer 4

Intro section, first sentence - if possible, please say a few words on what you mean by quality.

Reply:

Thank you for bringing this to our attention. We changed the first sentence to "In recent years, the methodological and reporting quality of biomedical research has been extensively discussed."

Second sentence - should be "In times when the mere..." Add the word 'the'?

Reply: Done.

You quote Doug Altman - but it is misspelled. Please add reference if there is one.

Reply:

Thank you, we corrected the sentence to "To maximise the benefit to society, you need to not just do research, but do it well." We added a reference.

'Some medical journals' are mentioned. Can you give examples: (e.g., Lancet, ...)

Reply:

Thank you, we added the following journals: "Some medical journals like JAMA, BMJ, PLOS Medicine, The Lancet, among others identified this problem several many years ago, …"

You mention reliability, perceived value, and reporting quality. Please define these briefly here. The EQUATOR network is mentioned. Please give a reference or website link.

Reply:

Thank you for pointing this out to us. We reworded the paragraph as follows:

"Simera and co-authors discussed the deficiencies in reporting medical research, making it difficult to assess "how the research was conducted" and to "evaluate the reliability of presented findings" [3], thus hampering their utilization in clinical practice. To maximize the value from funded research, so-called reporting guidelines

were developed to foster good research practices among clinical scientists, which are now organized within the EQUATOR network (www.equator-network.org)."

Methods section:
The frequency matching approach is mentioned. Please give a definition and/or reference.

Reply:

Thank you, we added the corresponding reference.

In the fifth paragraph, initials are used. I realized later these are for the authors. Please mention that here.
I liked how you identified the quality measures.

Reply:
Thank you, we added "the authors" before giving the initials to avoid any confusions.
